# Theorising the politics of famine: Bangladesh in 1974

**DOI:** 10.1111/disa.70041

**Published:** 2026-01-21

**Authors:** Naomi Hossain

**Affiliations:** ^1^ SOAS University of London United Kingdom

**Keywords:** Bangladesh, disaster, famine, food aid, international aid, mass starvation, politics of famine

## Abstract

1974 saw the first—and last—famine in independent Bangladesh. The disaster killed an estimated two per cent of the population and caused a crisis of legitimacy for the leadership of a nation that had won its independence only three years previously. Its catastrophic aftermath saw the emergence of an agreement among ruling elites and citizens that protection against mass starvation was a priority for the legitimation of political rule, or an ‘anti‐famine contract’. This article examines the event to revisit theories of the politics of famine at a time when episodes of mass starvation are on the rise. Utilising existing theories of famine politics, it establishes propositions about the conditions under which states have or acquire the political commitment and capacity to prevent or mitigate episodes of famine. The effort at theory building draws specific attention to how to incorporate the geopolitics of famine and humanitarian relief into the analysis of the political reasons why famines occur or are not prevented.

## 1 INTRODUCTION

The 1974 famine was the first and last episode of mass starvation in independent Bangladesh. It struck a new nation with a history of famines and other disasters resulting from its location in the Bay of Bengal and its vulnerability to global economic volatility. When the famine struck, poverty and malnutrition were chronic after centuries of extractive and disruptive colonial and neo‐colonial rule, exacerbated by the war of liberation from Pakistan in 1971. Disasters had shaped political history in the region: the liberation war was triggered in part by the callous response of Pakistani rulers to the 1970 Bhola Cyclone (Hossain, 2018), while the Indian independence struggle against British rule was energised by the 1943–44 Bengal famine (Mukherjee, 2011). By 1974, a pattern of political history had been established in which entrenched rulers lost legitimacy and faced opposition from failures to protect people against crises of subsistence and survival (Hossain, 2017).

This article has two aims. First, it seeks to analyse the politics of the 1974 famine. Although the event claimed the lives of around 1.5 million people (Alamgir, 1980), it has largely been ignored since its appearance as a case study in Amartya Sen's (1981) essay on the causes of famine. This paper reviews debates about the causes of the 1974 famine and explores its political effects. The aftermath saw the destruction of a regime that had seemed powerful and popular after it led the independence struggle; among its political effects were recognition that famine was politically costly. After 1974, systems to prevent and mitigate mass starvation were accorded a high priority, and Bangladesh has not seen episodes of mass starvation since (Hossain, 2017). The analysis presented here focuses on the national dimensions of the political incentives and state capacities to respond; a subsequent article will address the role of the international community, in light of theories that propose that famines may be the intended effects or collateral damage of a policy regime in which saving (some) lives is not a priority (Howe, 2006).

The second aim of this article is to contribute to famine theorising. Concentrating on the political aftermath of famines shows the emergence of what Alex de Waal (1996) has termed an ‘anti‐famine social contract’, binding commitments to prevent and protect against mass food insecurity. A key proposition here is that the elements of such a contract can be discerned in Bangladesh post 1974, including investment in and liberalisation of agriculture and agrarian markets, targeted social transfers, and early warning and foodgrain reserve systems, all relatively well‐resourced, authorised, and protected from undue political influence.^1^ These elements have broadly shaped public policies on food security and social protection since 1974 (Hossain, 2017).

The article is organised as follows. The next section reviews a selected range of theories on the political dimensions of famine, including radical approaches to understanding famine causation, Sen's entitlement theory and his assertions about democracy as a famine preventive, Dan Banik's specification of the Sen claims, critiques by Olivier Rubin, and de Waal's work on ‘anti‐famine social contracts’. De Waal's later work and Jenny Edkins' analysis of mass starvation as intentional political action are acutely relevant, as is David Keen's account of the ‘benefits of famine’. The application of selectorate theory to famine causation is also explored, including in relation to the role of international aid agencies. The subsequent section describes the 1974 famine in Bangladesh, providing a summary of arguments regarding its causes and its incidence. This is followed by an account of its political aftermath and ensuing reforms. A concluding section summarises the main arguments about the political effects of the Bangladesh famine, and the implications for famine theorising.

## 2 THEORIES OF THE POLITICS OF FAMINE

How can we understand why famines were prevented in Bangladesh after 1974? Common sense theories derived from Thomas Malthus's argument that ‘*population growth inevitably ends in gigantic famine*’ (de Waal, 2018, p. 38; original emphasis) treat famines as the natural result of a decline in the availability of food relative to population size, often triggered by a disaster owing to a natural hazard, ecological degradation, or conflict (Devereux, 1993). Bangladesh's large population experienced excess flooding in the wake of a devastating war in 1974, yet despite the presence of these key factors, Malthusian explanations are of limited help here. In the years since 1974, Bangladesh experienced major floods and cyclones and its population more than doubled. However, it also undertook significant investment in food security and disaster response, financed by external aid, creating space for international and domestic non‐state actors to work with impoverished groups, and largely insulated these policies from the vagaries of politics at home and abroad. Government legitimacy is broadly judged on its capacity to sustain and protect basic subsistence (Hossain, 2017). It is therefore necessary to understand the determinants of this intentional action to prevent famine after 1974, for which it is helpful to turn to theories of the political dimensions of famine.

This section sketches out some of the main claims of the different political approaches to famine. There have probably always been ‘political’ theories of famine, in the sense that situations of extreme widespread hunger are explained with reference to actions public authorities could take to prevent or mitigate the descent from dearth into starvation, or active policies—notably war and dispossession—that create the conditions for mass hunger (de Zwarte and del Arco Blanco, 2025). As Cormac Ó Gradá (2009, pp. 2–3) notes:

Although many observers in the past deemed them ‘inevitable’ or ‘natural’, throughout history the poor and the landless have protested and resisted at the approach of famines, which they considered to be caused by humans. The conviction that a more caring elite had the power and less rapacious trading class had the resources to mitigate – if not eradicate – disaster was usually present.

Louise Tilly (1983, p. 333) similarly notes:

[the] certainty of early modern English authorities that dearth and famine were caused by human acts, and could be prevented or corrected by public intervention. Their interpretation justified efforts to control merchants, markets, and the circulation of grain – a paternalistic role for local authorities in guaranteeing food at a fair price for ordinary people.

Famine theories indicate the direction of famine policies, as Ó Gradá and Tilly suggest. This may be why ‘political’ famine theories have been muted among the dominant official explanations of famines. Those tasked with accounting for such episodes were often themselves members of the classes or institutions fingered by political explanations, classes, and institutions whose ruling ideologies or interests were resistant to the claims of the hungry. For instance, Amrita Rangasami (1985b) shows how colonial administrators tasked with preparing India's Famine Commission reports could not help but uncover the role played by high prices, loss of livelihoods, and imperial tax and trade policies in propelling dearth into extreme hunger. Nevertheless, while administrators reported on the realities they observed, the official reports were ‘ambivalent’, ‘on the whole tended to exonerate government of blame’, and ‘tended to blame Nature not Man’ (Rangasami, 1985b, p. 1798).

### 2.1 The ‘new’ politics of famine

Significant interventions into debates about famine politics have addressed the ‘Nature not Man’ tendencies in famine analysis, challenging received historical wisdoms about famine causation, and sometimes even succeeding in inserting anti‐Malthusian thinking into key policy domains. Brazilian geographer, diplomat, and activist Josué de Castro is remembered for his ‘geography of hunger’ analysis, first developed in the city of Recife and then extended globally. De Castro's work established spatially‐situated, socio‐political explanations of local hunger rooted in patterns of colonial domination and dispossession and capitalist exploitation of people and place. Hunger, he argued, was a powerful tool with which to advance the demands of empire and capital: ‘it is through hunger that the *latifundia* [plantation] proceeds’ (de Castro, cited in Davies, 2023, p. 3). Although de Castro's work has left few clear traces in contemporary theorising of famine, his ideas were influential at the Food and Agriculture Organization of the United Nations (UN) in the 1950s as a counter to the ‘dogma of scarcity’ and the heft of Malthusian arguments following United States President Harry S. Truman's ‘invention of underdevelopment’ in 1949 (Ferretti, 2021, p. 597).

Yet even popular and influential counter‐Malthusian explanations like that of de Castro failed to destroy what de Waal (2018) terms the ‘zombie’ theory that famine results from overpopulation. Mainstream understandings of the causes of famine into the 1960s and 1970s drew on reheated Malthusian claims about the supposedly growing gap between global food production and population growth, popularised by bestsellers such as *Famine 1975! America's Decision: Who Will Survive?* (Paddock and Paddock, 1967) and *The Population Bomb* (Ehrlich, 1968). These ideas shaped both ‘common sense’ and policy perspectives on famines and on how and indeed whether they should be addressed (United States Congress, 1975).

It was in this context that Sen's (1981) *Poverty and Famine: An Essay on Entitlement and Deprivation* broke new ground, challenging the enduring and influential view that famines were caused by a decline in the food supply. His theory of entitlement failure focuses on what food people are legitimately (according to law and custom) entitled to on the basis of what they can earn, exchange, or elicit as gifts: ‘starvation [results] from a failure to be entitled to a bundle with enough food’ (Sen, 1981, p. 45). Sen's famous essay helped edge policymakers' understanding of famine away from ecological and Malthusian explanations and towards analysis of the reasons why people lack or lose their ability to access sufficient food (Devereux, 2006b; Rubin, 2019).

Sen's theory synthesised insights from history and the public administration of famine into a legible account of how public policy made—or could be designed to prevent—famines. While enabling a more precise description of the mechanisms through which individual people come to be in a state of starvation, the ‘entitlement theory of famine’ elicited a series of critical responses that in turn articulated a clearer and more intentional role for politics and power in the making of famine. These noted that Sen's theory failed to account for the relational and structural factors that push populations into such a condition in the first place, and which prevent their liberation from it. In a pair of influential essays, Rangasami (1985a, 1985b) pointed out that Sen's conception of famine started at what close analysis indicates should be seen as its endpoint, the point at which people visibly starve *en masse*. This limited focus means that it failed to account for the factors that create (i) dearth (a condition of shortages and/or high prices of food), which unaddressed, leads to (ii) increasing levels of hunger or ‘famishment’, and from there to (iii) mass morbidity; this final stage is the point at which Sen's inquiry begins (Rangasami, 1985b). Drawing on evidence from her own investigations of hunger across contemporary India, during the Dutch famine of the Second World War, and in the Indian imperial Famine Commission reports, Rangasami (1985a, p. 1750) defines famine as:

a process during which pressure or force (economic, military, political, social, psychological) is exerted upon the victim community, gradually increasing in intensity until the stricken are deprived of all assets including the ability to labour.

This redefinition of famine demands the identification of ‘the various factors, political, social, psychological and economic that operate to keep large classes in the population under a continuous pressure’ (Rangasami, 1985b, p. 1801). This ‘pressure or force’ cannot be accounted for by entitlement theory because of its methodological individualism. It ‘privileges the economic aspects of famine and excludes the social and the political’, including the institutions that determine entitlements, the disruption and social breakdown entailed, and the violence and dispossession involved in the complex political emergencies associated with famines in the late twentieth and early twenty‐first centuries (Devereux, 2001, p. 259).

Sen's (1983, p. 334) entitlement theory drew attention to ‘the economic, social, and political relationships of those who suffer, and die, in famine’, arguably a Trojan horse to smuggle in political explanations for famine under the economistic analysis of the mechanisms through which dearth turns into starvation. While ‘common sense’ thinking may still favour overpopulation as an explanation of famine, following Sen's attack on the assumption of food availability decline, policy debates about famine took a political turn, reflecting the ‘complex political emergencies’ of ecological collapse, decimated livelihoods, violent conflict, and low levels of state capacity that characterised recent famines (Devereux, 2006a). These theoretical shifts have treated politics as central, with an ‘analytical focus on failures to prevent famine, rather than on the triggers of food shortage or disrupted access to food’ (Devereux, 2006a, p. 7).

### 2.2 Democracy and anti‐famine social contracts

The question of whether democratic institutions act as a break on the descent from dearth into catastrophe gained salience as the evidence of China's Great Leap Forward famine (1958–61) emerged in the 1980s. Very likely the worst in world history, with mortality estimates ranging in the tens of millions, the famine was a clear case of a decline in the availability of food originating in disruptive agricultural collectivisation policies. In *Tombstone*, Yang Jisheng (2012) lists a range of political factors that provided the systemic causes of the disaster: Chinese Communist Party Chairman Mao Zedong's imperial mode of governance, the state's control of the economy and the means of production, the totalitarian leadership of the Chinese Communist Party, enforced through violence and tight social control, and the system's inability to correct errors of governance. The absence of mechanisms for holding the leadership to account, along with acute pressures on lower‐level officials to report optimistic agricultural growth, created the conditions for concealment of the famine on a vast scale (Wemheuer, 2010; Yang, 2012).

Jean Drèze and Sen (1991) explore the contrast between the major Chinese and Bengal famines of the mid‐twentieth century, notably the fact that the famine in China continued for several years without apparent official recognition or policy response. The contrast between the two cases—totalitarian communist China versus the mixed economy and noisy democracy of India—appear to have encouraged Sen's other contribution to theories of famine politics: the proposition that famines do not occur in democracies with a free press. This contribution has had a more chequered career than entitlement theory (Devereux, 2001; Rubin, 2009, 2011; Burchi, 2011). It has spawned multiple arguments and empirical tests, including Banik's (2007) study of the political and institutional determinants of starvation in democratic India and Rubin's (2008, 2009, 2011) cross‐country comparative analysis. Results from both suggest mixed but weak support for the proposition that democracy and a free press are sufficient to deter famine, or even that they make famine less likely. One reason is that democratic institutions do not necessarily generate strong incentives to prevent or mitigate starvation. In the Indian state of Orissa (now Odisha), incoming governments blamed the previous party and concealed evidence of hunger on their own watch, while local collective action was too weak to make effective demands on an also weak local government system (Banik, 2007). Democratic competition can actually hinder famine relief and prevention. Citing Paul Brass's account of the Bihar famine in the 1960s, Rubin (2009, p. 705) points out that:

[T]he ‘Bihar Famine crisis was not only politicized from its onset, but it was democratized.’ … many different voices in the democratic process – the free press, the citizens and the opposition parties – all had a say in defining the situation… Questions such as whether or not there was a famine; who should intervene to prevent the famine; and who should bear the responsibility for the famine were the focus of a political struggle ultimately leading to a suboptimal response.

Multiparty democracy may not endow states with the capacities and resources to respond, even where the political incentives are present (Rubin, 2008). Malawi, at the time of the 2002 famine, was an electoral democracy with a free press exerting pressure on political discourse and creating incentives for politicians to act. It was also a poor country with a significant problem of hunger and malnutrition, which was highly dependent on aid donors who were pushing for liberalising reforms of the food system. In 2002, donors failed to support Malawi's relief system, and several key policies squeezed its public finances (Rubin, 2008; see also Devereux and Tiba, 2006). Banik (2007) similarly finds that institutions mandated to deliver nutritional and emergency assistance were under‐resourced and faced limited pressure to respond to the hunger crisis in Orissa: administrative procedures were not operational, the bureaucracy was unmotivated, and judicial and quasi‐judicial interventions lacked teeth. The latter case illustrates a situation in which mixed political incentives to declare and address famine were matched by a weak and uncoordinated administrative system.

Democratic institutions and a free press also had little impact on chronic hunger in Malawi and India: if pressure on governments to prevent hunger depends on the shock value of media coverage of starvation, chronic hunger may not qualify as ‘news’ at all. Yet, extreme poverty and chronic hunger make episodes of mass starvation more likely (Currey, 1978, 1992; Rangasami, 1985a).

These advances on Sen's democracy–famine proposition have methodological implications. Rubin's important body of work concludes that the democracy–famine proposition does not hold: democratic competition can be adverse and authoritarian rule positive for famine prevention and mitigation, as China after the Great Leap Forward famine shows. Political incentives and institutional capacities to prevent or mitigate famine are not reducible to regime type; rather, they are distributed in different ways and to different degrees across political systems. The complexity and variety of famine means that case‐specific analysis is necessary to theorise from specific famine episodes (Rubin, 2009). Banik's (2007) fine‐grained analysis of the politics of hunger in India points to the institutions and centre–local dynamics shaping the capacities and incentives to act to understand how the exercise of political power influences efforts to address famine.

De Waal's (1996) concept of an ‘anti‐famine social contract’ takes up the challenge of investigating political incentives and institutional behaviours directly, drawing attention to the political features of sustained efforts to prevent famine. Examining the emergence of the famine codes and the post‐independence approach to famine in India, he argues that ‘[f]amine prevention is intimately bound up with the entire ideology of Indian nationalism’ (de Waal, 1996, p. 196). Historical political struggles are necessary to establish such a contract, but its enforcement depends on whether and how rulers recognise that it is a ‘political necessity’ to respond:

The basic reason why a government prevents famine is because its interests – the power of its leaders – depends on it. There is a political incentive to prevent famine. Elected politicians fear the retribution of their constituents in the polling booths, and hope for the electoral reward of successfully delivering famine prevention. Civil servants fear disgrace or demotion of (sic) their failure to prevent famine is exposed (de Waal, 2000, p. 13).

De Waal (2018) develops this thinking further in relation to the political responses to famine in Ethiopia in the late twentieth and early twenty‐first centuries. The lessons of the crises of 1973–74 and 1984 included that famines threatened national security and political elite survival: ‘One of the sparks for the 1974 revolution had been that the emperor failed to stop a drought turning into a famine and then denied the seriousness of the famine’ (de Waal, 2018, p. 150). The party that wrested power from the Dergue in 1991, the Ethiopian People's Revolutionary Democratic Front (EPRDF), saw famine prevention as both a strategy for building its legitimacy and support with its peasant base, and a means of bolstering national security, which had historically been threatened by insurgencies and rebellions arising out of episodes of mass hunger. In addition, it boosted agricultural investment and introduced a vast social protection scheme, the Ethiopia Productive Safety Net Programme, information systems to detect food crisis and allocate relief, and, latterly, drought insurance, as part of the strategy for tackling acute hunger (de Waal, 2018).

These policies continued to help prevent the recurrence of mass starvation in Ethiopia despite democratic backsliding from 2005 onwards; a period of rising repression and a crackdown on civil society and the media also saw a remarkably successful relief programme in 2015–16, reflecting the fact that it was ‘a strategic political commitment, not a liberal‐democratic sensibility’ (de Waal, 2018, p. 151). In 2018, de Waal noted that the commitment to famine prevention did not apply to all Ethiopians, and that military strategy continued to utilise famine against insurgents when it saw fit; the commitment had not yet become ‘a genuine social‐political contract with the citizens [for which it needed to be] part of a democratic process’ (de Waal, 2018, p. 153). De Waal's assessment of the limited durability of the EPRDF's anti‐famine ‘contract’ based on political strategy rather than a democratic social contract was borne out by events shortly after his book was published. In 2018, Abiy Ahmed's Prosperity Party replaced the EPRDF, and by 2020–21, was using famine as a weapon of war against the region of Tigray (Weldemichel, 2022).

### 2.3 From policy failure to priority regime

The assumption that states are motivated to prevent famine has itself been challenged, and there is growing analysis of the conditions under which states and transnational bodies actively or passively create the conditions for dearth and its descent into mass starvation. Edkins' (2002) work is particularly important here in arguing against ‘technical’ understandings of famine, noting that they are always the result of political choices and practices which warrant close investigation. She is critical of Sen's treatment of states as essentially benign liberal institutions, and, in agreement with de Waal, argues for the treatment of mass starvation as a criminal act (Edkins, 2002, 2006; see also de Waal, 2018). A key point is that, with the technical means of supplying food aid to starving populations available in the present day, famines are properly understood not as failures of social and economic systems and the policy regimes that govern them, but as their products, so that merely improving technical approaches to famine response cannot succeed in preventing mass starvation:

famine is a product of power relations. It is not a question of finding better early warning systems, more participatory development projects, or faster methods of delivering relief. Nor is it a question of seeking the deeper, more structural causes of famines, nor its complexities. Famine is a product of violence. Even where war is not implicated directly, the state enforces laws of property that can lead to some people's starvation. Aid processes and interventions to which technical concepts of famine give rise are practices that reproduce particular political and international power relations (Edkins, 2000, p. 156).

The treatment of politics and power as intrinsic to explanations of famine pays close attention to the role of capitalism and empire. Michael Watts' (1983) account of subsistence crises in nineteenth and twentieth century northern Nigeria built on de Castro's spatial, historical and institutional methodological approach to show how the peasant ‘moral economy’ had been eroded by the disruptions wrought by capitalist modes of agrarian production and exchange. These left them acutely vulnerable to the volatilities of climate and markets (Watts, 1983). Mike Davis's (2001) epic account of the *Late Victorian Holocausts*, the El Niño famines of the late nineteenth century, clarifies the role of imperial policies in dispossessing populations, disrupting and expropriating local economies, imposing unviable taxes and tariffs on peasants, and failing or refusing to manage dearth or to mitigate mass starvation. The late nineteenth century droughts were so lethal in part because of the actions and the inactions of imperial powers, steeped in Malthusian ideology and colonial racial hierarchies (Davis, 2001).

David Nally's (2008) work on the Irish ‘potato blight’ famine of the 1840s applies an analysis of ‘colonial biopolitics’ to help explain the failure of the British response to ameliorate or prevent the years of devastation. He follows Edkins in using Michel Foucault's concept of biopolitics, or ‘the state‐led management of life, death, and biological being—a form of politics that places human life at the very center of its calculations’ to explain why (some) Irish lives were deemed expendable or beyond saving. This produced an experimental approach to policy interventions during the famine, in which starving Irish peasants were the guinea pigs (Nally, 2008, p. 716).

Following Edkins in moving away from ‘technical’ approaches to famine which treat them as policy failures, famines may be seen as part of broader processes of dispossession and conflict that benefit some groups (Keen, 2008), and not ‘failures’ of policy at all. Keen's (2008) study of the Sudanese famine of the 1980s showed how central government in the north, military officials, and local political actors pursued political and economic gains from mass displacement and loss of livelihoods. At the same time, the distribution of humanitarian relief was disconnected from those in greatest need, was politicised, and ultimately benefited powerful groups (Keen, 2008).

Drawing on case studies of the international and domestic responses to recent famines, Paul Howe (2006) develops the ‘priority regime’ approach to integrate key political policymaking dimensions into the typically technocratic international community approach to famine. He identifies a range of types of political choices in how famines are prevented, enabled, mitigated, or neglected. Priority regimes may have a range of different relationships to famine, including neglect (when the possibility of famine is ignored or not considered) or occurring as a byproduct of another, higher priority policy or as the result of a trade‐off for a higher priority policy; famine may also be a means towards achieving some other goal, or be the goal itself; lastly, the priority regime may be actively trying to prevent or mitigate mass starvation (Howe, 2006; see also Howe, 2018).

Another approach to theorising the politics of famine which ends up in a similar place to Edkins, Keen, and Howe (albeit from a very different theoretical starting point) is the application of selectorate theory to explain why governments might fail to act to prevent or mitigate famine. Thomas Plümper and Eric Neumayer (2009, p. 50) show that:

governmental inaction in the face of a severe famine threat can be the rational outcome of a political support maximization calculus. Governments may rationally fail to act against famines when the political costs of action are higher than the political costs of inaction.

Plümper and Neumayer (2009) note that protecting some groups is more politically salient, and this is one reason why democracy does not necessarily protect against famine. Both democracies and autocracies may choose rational inaction, depending on whether potential famine victims are key segments of their support base. International food aid can help relieve pressure on government relief efforts, making the policy choice to help even non‐members of the selectorate a viable option. However, the democratic pressures of electoral and popular legitimacy mean that autocracies are more likely than democracies to allocate food aid to key constituencies rather than those who need it most. An unhappily good example of this is the 1984 Ethiopian famine, well‐known in the Global North because of the star‐studded Band Aid concerts which tried to raise funds and awareness, ushering in a new era of celebrity humanitarianism. De Waal (1997) notes that the main cause of the 1984 famine was the war the Ethiopian government was fighting against insurgents in Tigray and Wollo between 1980 and 1985. The worst effects of the famine were felt by the people in these regions, punished in effect for being from a place in which insurgents were active. The government prevented people from moving away from active warfare, causing prices to rise. It also forced some people to move south, leading to the deaths of at least 50,000 people. And it manipulated relief, to ensure its supporters rather than people in the areas of its opponents benefited. The international community, the UN, and humanitarian relief agencies and charities were unwittingly—and sometimes knowingly—complicit:

This relief programme supported President Mengistu militarily and politically. In Tigray, very few rural people and very many soldiers were fed by the relief. The humanitarian effort prolonged the war, and with it human suffering (de Waal, 1997, p. 127).

### 2.4 Incentives and capacities to mitigate or prevent famine

These contributions help clarify key propositions about why governments fail to mitigate or prevent famine. They can be summarised as political incentives and institutional capacities respectively either to mitigate (in the short term) or prevent (in the medium term) episodes of mass hunger. I do not here address in detail a third potential category of political actions, namely the famicide or active promotion of famine as identified by Howe among others, although the politics of promoting famine are increasingly evident and important (Keen, 2008). The aid blockade that created the famine in Gaza (2024–25) is a particularly clear example of a deliberate intervention designed to starve a population (de Waal, 2024; Devereux, 2024). This third potential category of political actions to create or promote famine will be assessed in a subsequent paper.

The literature on the politics of famine causation and prevention identifies three levels at which these political incentives and institutional capacities are likely to operate:

1. National—where core political competition is staged, policies get made and implemented, and aid relations negotiated.
2. Sub‐national—where policies are implemented with national oversight and government meets citizens on the frontlines.
3. International—where aid relations are negotiated, and which authorise and resource the implementation of aid programmes, both emergency and developmental. Dependence on humanitarian aid means the political incentives and institutional capacities to mitigate famine in the short term or prevent it in the medium term are shaped by considerations at the level of the international community, in particular aid relations. The recent withdrawal and shrinkage of traditional overseas development assistance and the rising significance of the BRICS (originally Brazil, Russia, India, China, and South Africa) countries and the Middle East as aid and humanitarian aid donors is likely to change the nature of the policy space and aid conditions in question.

A non‐exhaustive list of potential political incentives and features of institutional capacity which help to explain why governments may act to mitigate famine through effective relief or emergency measures in the short term may include the following:

1. National political incentives are likely to include the expected political costs of failing to act: losing elections and legitimacy; vulnerability to political challengers; and the threat of unrest. Positive incentives may include receipt of aid; but flows of food aid may also create incentives for corruption. Having the institutional capacity to respond implies information and early warning systems, foodgrain reserves, and administrative capacity to distribute relief. Policies of suppressing minority or secessionist rights claims may also shape efforts to tackle famine, as well as actively to promote it among those groups.
2. Sub‐nationally, the political incentives to act may include accountability to the centre, which in turn depends on effective oversight and management at the national level, as well as information. Localised conflict and tension between different ethnic or religious groups may reduce the commitment to fair distribution of famine relief.
3. Internationally, the political incentives to support emergency famine relief may result from international law and policy frameworks, the desire to maintain bilateral relations with the famine‐affected country, and to be seen to be trying to help starving people. International actors, though, may lack the capacity to supply or finance food aid, or the necessary aid relations through which to facilitate transfers and assistance.

The political incentives to prevent famine through building food security for the medium term are likely to be similar at each level, with accelerating development and economic growth an additional factor. The institutional capacities to prevent famine in the medium term differ from those needed to deliver effective relief, however, involving longer‐term technical support for investments in food systems more broadly. Prevention strategies require a degree of stability and level of overall development that may not be present in conflict settings, as found in recent famines.

For aid‐dependent developing countries, we would expect that in the short term, incentives and capacities to deliver food aid to the hungry would need to be present at all three levels (national, sub‐national, and international) for famine to be effectively mitigated. These incentives and capacities do not exist independently of each other: if incentives are sufficiently strong, national political elites may draw on their political capital to enjoin local elites to deliver aid or make commitments to international donors to attract emergency aid. Ultimately, and again drawing on the insights of Rubin, de Waal, and the selectorate famine theorists, we would expect national political commitment to mitigate (or prevent) famine to be the single most important factor, and the source of incentives and capacities at other levels. Yet, in the absence of other conditions, national political commitment is unlikely to be sufficient to mitigate a famine which has already reached the stage of mass starvation.

Famine prevention is a longer‐term project, and we can expect that where the political incentives exist, institutional capacity may be built or strengthened over time to prevent famine. Ultimately, however, aid‐dependent developing countries may need to reduce their reliance on emergency aid, that is, at the international level to be assured of national food security in the medium to long term. This is particularly true since 2025, when development and humanitarian aid are facing steep cuts as the Global North withdraws from its earlier commitments.

The next section describes how the 1974 famine unfolded in Bangladesh. It is followed by an analytical discussion, applying the proposed framework to the evidence provided.

## 3 THE CAUSES OF THE 1974 FAMINE IN BANGLADESH

By 1974, the country that was now Bangladesh had experienced a deadly cyclone that killed perhaps 500,000 people (occurring four years earlier) and a bloody conflict marked by mass killings and rapes and secession from Pakistan (ending two years previously). After centuries of colonial and neo‐colonial rule, a large majority of the population were undernourished; a growing proportion of rural people were landless labourers; and the administration and the economy had been weakened by the conflict, the secession, and the weak and corrupt government. A global oil and food price crisis added to the emergency context. Against this backdrop, four broad explanations of the 1974 famine have emerged in the literature: floods; entitlement economics; the politics of international food aid; and the famine relief distribution system.

### 3.1 Floods

The 1974 floods were unusually destructive (Currey, 1978), and followed the 1970 cyclone, the 1971 liberation war, and the OPEC (Organization of the Petroleum Exporting Countries) oil price crisis. A majority of Bangladeshis had been under severe livelihood and nutritional stress for years by the time food prices spiked in August (Franda, 1981). The floods played a specific role in tipping millions from stress into disaster:

[T]here is no denying that the flood accentuated human suffering that was already in evidence, and all those families who were living well below poverty level, finally succumbed to the pressure. By the end of July, the scenario in all flood‐affected regions of the country, was flood leading to loss of human and cattle life, loss of agricultural land and crops, loss of homestead, and loss of employment, all of which combined to lead to starvation and outbreak of epidemic diseases (particularly cholera) (Alamgir, 1980, pp. 126–127).

Bangladesh was facing a macroeconomic crisis and food supplies were scarce. Aid flows helped fill the gap, but only while the floods were visible, and ‘observers formed the impression that the government was “crying wolf” and exaggerating the flood damage’ (Crow, 1984, p. 1757). Another flood event hit northern districts in September, by which time disease and hunger were widespread. In September, the government made an emergency appeal for food aid, declared a famine, and opened 4,300 *langarkhanas* (feeding camps) to feed up to three million people per day.

### 3.2 Entitlement economics

As the floods rolled out, the government calculated the food gap at three million tons (total foodgrain production was around 12 million tons of rice and wheat) (Alamgir, 1980, p. 129), but the extent to which low food supplies caused the famine remains contentious. Production was not lowest in the worst‐affected regions, nor lower overall than in previous years (Sen, 1981, p. 141). But a broken transport system, fears of forcible procurement, and smuggling to India meant local supplies were erratic and uneven, and a decline in foodgrain availability could not be ruled out entirely (Alamgir, 1980, p. 239).

There is more agreement that famine occurred because people had no entitlements to the food available. The floods meant less work harvesting crops, and alongside new technologies, declining jute export earnings, and rapid rural population growth they resulted in the agricultural wage declining by 43 per cent between 1973 and 1974 (Alamgir, 1980, p. 311). The material endowments of Bangladeshis had been depleted over so many years that the floods pushed people into the abyss:

[E]ntitlement failure in 1974 turned out to be as precipitous as it was mainly because of severe entitlement contraction that had occurred in the preceding years, partly through natural calamities and partly through the destructions and dislocations caused by a prolonged war of liberation. The destruction of assets (houses, cattle, etc) caused by these events was a direct dent in the ‘endowment set’, especially for the rural people. Endowment contractions of this kind must have accentuated the gravity of the famine (Osmani, 1991, p. 332).

**FIGURE 1:**
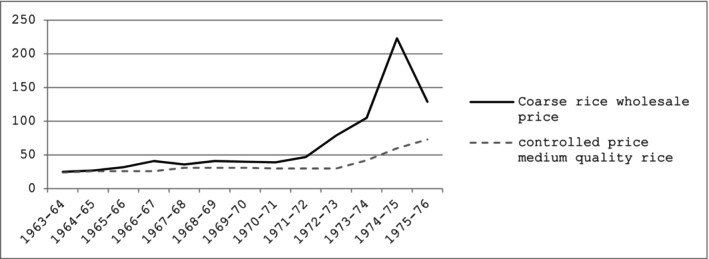
Rice prices from 1963–76 (per maund, Bangladeshi taka). **Source:** author's calculations based on Alamgir (1980, p. 260, Table 7.1).

Coarse rice prices increased four‐fold between 1971 and their peak in 1974 (see Figure 1) (Alamgir, 1980, p. 260). Why prices spiked in 1974 is another matter of debate. There is some evidence that speculative hoarding was at work, as the government was believed to be in no position to stabilise prices after the damage predicted owing to the floods (Ravallion, 1985). Grain traders assumed prices would rise because of predicted crop damage (Ravallion, 1985; Quddus and Becker, 2000; Del Ninno, Dorosh, and Smith, 2003); they were also aware that government stocks were considerably lower than the level that would signal an impending food crisis (Crow, 1984). This led to ‘the collapse of confidence in the government's ability to stabilise the price situation in the coming months’, and to highly lucrative stockpiling (Islam, 2003, p. 223). As Ravallion (1985) notes, evidence of the extent of hoarding is at best, anecdotal, although accounts of storehouses being raided by militant leftist and peasant groups circulate from the period (Hossain, 2017). The extent to which smuggling mattered in relation to the spike in prices is less clear; theory‐based economic analysis at the time concluded that smuggling was unlikely to have been on a sufficiently large scale to have caused the fluctuations (Islam, 2003). However, popular perceptions and those of the army officials tasked with policing the grain trade were at odds with the sanguine analysis of the economists. The popular belief was somewhat vindicated in the aftermath of Prime Minister Sheikh Mujib's assassination in August 1975: food prices dropped sharply, presumably because grain traders and dealers believed they had lost their political protection, and dumped stocks accordingly (McHenry and Bird, 1977).

### 3.3 The politics of international food aid

Food aid was vital for the Bangladesh economy and government spending (Clay, 1979). By mid‐1973, the government was having difficulties securing the necessary commitments because of global commodity spikes and its own corruption and mismanagement (Atwood et al., 2000). World food prices were high and the government bankrupt, so commercial imports were unviable (Rothschild, 1976). Because Bangladesh had sold Cuba jute sacks worth USD 5 million, US PL480 (Public Law 480) food aid was withheld on grounds that recipients could not trade with communist countries. Even when the Bangladesh government cancelled future trade, US government lawyers stalled; food aid was only agreed a year after the initial request (Islam, 2003). ‘This grim drama’, recalls Rehman Sobhan (1979, p. 1979), ‘was being transacted in the capital city in the full view and knowledge of the US embassy as to the nature and gravity of the crisis’.

Why did the US government delay food aid? For Sobhan (1991, p. 104), the Cuba–PL480 crisis was an attempt to call Bangladesh to heel:

Its decision to suspend PL‐480 shipments to Bangladesh against pre‐committed food aid on grounds of Bangladesh's miniscule exports of jute goods to Cuba should thus be seen not just as an attempt to constrain the regime's external relations, but as a direct assault on the viability of the regime. The political motivation underlying this embargo may be derived from the fact that at that time [US Secretary of State Henry] Kissinger sought a special dispensation for [President Anwar] Sadat's Egypt to continue exporting cotton to Cuba while receiving US PL‐480 commodity aid.

Certainly, the US government deployed food aid to wield power over bankrupt, hungry Bangladesh (McHenry and Bird, 1977), but geo‐strategically unimportant Bangladesh was also of low priority (Rothschild, 1976). The crude but then popular ideology of ‘triage’—that resources should be concentrated on those likely to survive rather than those destined not to—was also among the ideological baggage with which the US government addressed the Bangladesh famine (Rothschild, 1976; Tweeten, 2001; Hossain, 2021).

### 3.4 The famine relief distribution system

The failure to provide adequate relief is the reason Mujib's government is blamed for the famine. People were dying in the streets of Dhaka by August, weeks before the official famine declaration:

By the end of August, the whole of Bangladesh turned into an agonizing spectacle of confusion and human suffering. With the addition of the flood, it was 1943 re‐enacted. Streams of hungry people (men, women and children), who were nothing but skeletons, trekked into towns in search of food. Most of them were half‐naked. Events such as husbands deserting wives and children, or wives doing the same, parents trying to sell children, mothers killing babies out of frustration and anguish, man and dog fighting for a piece of bone, women and young girls turning to prostitution became very commonplace (Alamgir, 1980, pp. 128–29).

By September 1974, there was a 14‐fold increase in unclaimed corpses on the streets of the capital (Currey, 1978).

The government set up 5,792 *langarkhanas* across the country, which at their peak fed 4.35 million people, six per cent of the population. Allocations were meagre, with some offering bread worth only two or three hundred calories each day; corruption was widely reported. The *langarkhanas* drew the hungry in, but failed to nourish or protect them against disease:

[U]tterly inadequate quantities of eatables or semi‐eatables are distributed to hundreds of men, women and children by persons with lathis (sticks) in their hands… In the unions of Rangpur, I have seen langarkhanas where hundreds of starving people came from four, five, six miles afar and wait all day, finally to get only one chapatti (hand made bread) the weight of which varies according to daily relative supply of Ata (wheat flour) … the journey to and from the langarkhanas back to the village may cost more calories than the langarkhana supplies (Anisur Rahman, cited in Alamgir, 1980, p. 176).

The Public Food Distribution System was the single greatest obstacle to effective emergency relief. The system was massive, covering 20 million Bangladeshis by 1977 (World Bank, 1977), but the main channel, Statutory Rationing, benefited better‐off urban people, public sector workers, and ‘priority groups’, while Modified Rationing distributed the residual to some of the rural poor. Urban bias, corruption, and leakage meant that few of the rural poor ever saw this residual (Chowdhury, 1988), and Statutory Rationing was never diverted towards famine relief (Sobhan, 1979). In 1974, nearly 60 per cent of rations went to urban people, although less than nine per cent of the population was urban (Muqtada, 1981).^2^ All of the urban population was officially covered during 1973–75, as compared to only six per cent of rural people (Chowdhury, 1986).

Why did the government fail to reallocate foodgrains towards relief? By September 1974, the rural poor were visibly starving *en masse*. Mujib's speech at the UN on 25 September 1974 acknowledged the situation:

Bangladesh, which was born on the ruins of a devastating war, has, ever since liberation, been plagued by a series of natural disasters, the latest one being the unprecedented floods we have experienced this year… These natural calamities not only have impeded the march of Bangladesh towards economic progress, but have also left the country in a state of near‐famine conditions.^3^

Mujib acknowledged that 27,000 people had starved to death, a figure from which the government appears to have taken comfort:

Everybody expected that few millions will die by starvation, after the flood and after the inflation… And round about 27,000 people have died by starvation. Which is a fact. We have tried our best, consciously we have tried our best (Pilger, 1975).

Any recently elected government would have consciously “tried their best” to prevent such a catastrophe: the 1943 famine is often seen as having sealed the fate of the British Raj and factored in the politics of partition (Bose, 1990; Mukerjee, 2010), and the casual response to the 1970 Bhola Cyclone spurred the liberation war. But reallocating rations may have been administratively infeasible (Maniruzzaman, 1975b). By September 1974, it was too late to stabilise prices or distribute relief through rationing channels: the administrative challenge had become to feed the hundreds of thousands displaced by hunger. The civil administration was not equipped, nor the army co‐opted for the task. Although the UN's post‐war office continued to operate in Dhaka, the government was substantially on its own in dealing with this new catastrophe (Hossain, 2017).

Even if the administrative capacity had been present, there was no political will to divert foodgrains away from the urban and middle classes; this was deemed ‘too politically hazardous for a regime already under severe political attack for the sharp escalation in prices’ (Sobhan, 1991, p. 101). The starving rural poor were no political threat, but urban and middle class groups were. Leftist opposition groups and factions within the party were challenging the Awami League's leadership and food security was an explicit concern (Maniruzzaman, 1975b). And it was not only the poorest who were affected: the industrial workforce, low paid public sector workers, and even the middle classes struggled to meet the rising living costs (Sobhan, 1979).

The politicisation of food security may have inadvertently drawn political attention away from the masses, fixing it on politically active urban groups. Rationing formed part of the overall package of public service benefits, ‘the means by which subsistence wage goods were guaranteed to politically essential elements of society and government, through a period of increasing instability of supply and rapid price inflation’ (Clay, 1979, p. 130; see also Rashiduzzaman, 1977, p. 795). From a political economy perspective, the protection of Statutory Rationing through the famine period was part of a structural class bias operationalised in the form of rations for the urban and middle classes (Franda, 1981; Chowdhury, 1986, 1988). That the Awami League government put in place structures to prioritise the middle classes was consistent with its support base. Food aid provided the resources on which this political settlement briefly rested, financing a significant portion of the public sector benefit package at the time, and helping the Dhaka government appease the politically important urban and middle class groups. And so, food aid was not reallocated to shield the rural masses from starvation.

### 3.5 Analysis of the failure to mitigate famine in the short term

Returning to the discussion about why governments fail to mitigate famine, it seems that, in this case, at the national level, the political leadership had strong political incentives to deliver effective famine relief: it knew failure to do so would be costly for its legitimacy, in terms of the prospects for further unrest, challenges from the left, and (until it installed a one‐party state) potential electoral defeat. Warning systems were in place and the authorities were aware that famine was coming, but the post‐war administrative system was weak and demoralised and foodgrain reserves were inadequate. The political incentives to supply relief to the starving were weaker, however, than those to protect the vocal and politically organised urban and middle‐class populations, to whom most food aid was allocated. Despite the urgent situation and collective political costs, political leaders hoarded and stole relief goods, testifying to the weakness of Mujib's political authority.

Sub‐nationally, there were weaker political incentives to act, and those that did influence relief management related to local security. Rent‐seeking is believed to have occurred in the form of corruption in the *langarkhanas*.

Internationally, the major food aid donor, the US, was disinclined to help. It pointed to legal restrictions against aiding the famine effort, but ideological issues likely played a role: Bangladeshi policy was not aligned to the West, and ‘triage’ theories influenced the policy response to geopolitically‐insignificant Bangladesh. It is also the case that compared to earlier periods, US food aid was running relatively short due to the 1973–74 OPEC crisis, and thus internationally, too, institutional capacity to share food aid was comparatively limited.

## 4 THE EFFECTS OF THE FAMINE

While little has been written about the political effects and aftermath of the famine in Bangladesh in 1974, there is tacit consensus that it was a turning point in elite recognition of the sources of political legitimacy:

[T]he occurrence of a famine so soon after independence caused a massive crisis of legitimacy for the then government whose violent overthrow a year later was seen as an expression of the loss of this legitimacy. The crisis of legitimacy due to a failure to contain the famine appears to have become for subsequent governments a crucial political concern (Rahman, 1995, p. 278).

The famine was followed by sharp reversals in food security policy aimed at preventing food crises, suggesting institutional learning and cognitive change among the policy elite that triggered individual, political, and organisational trajectories of wider national importance.

### 4.1 Political upheaval after the famine

Famine has often delegitimised ruling groups, affecting the balance of political power. In wartime Bengal, the Communist Party's failure to focus on famine relief lost it support in East Bengal after the war, whereas the Muslim League's support for starving peasants contributed to its electoral success (Bose, 1990, p. 725):

The two parties which were relatively free to operate—the Muslim League and the Communist Party—were collaborating with the British which precluded political organization of famine victims. The Communists were anxious to avoid class conflict and were preaching the virtues of peasant–landlord ‘friendship’. While the Muslim League ministry in the province remained cautious, conservative and subservient to the Governor and the colonial bureaucracy, the League party organization under a new radical leadership from the winter of 1943 began to take a clearer anti‐landlord and anti‐grain merchant stand. The memory of famine victimization in no uncertain way contributed to the League's impressive victory in the post‐war provincial elections; it was also a potent ingredient in the reprisals taken by largely Muslim peasant and landless labour against mostly Hindu petty rent‐collectors and grasping grain‐dealers in the Noakhali and Tippera riots of 1946.

By contrast, during the Indochina famine of 1945, the nationalist Viet Minh died alongside the Tonkin peasantry, and their strategy of seizing granaries to feed the starving helped build their support in the north (Bose, 1990). In Ethiopia, armed peasant groups fought long‐running campaigns against the state following the 1973 famine, which is widely understood to have led to the downfall of the regime of Emperor Haile Selassie, not because hungry peasants and nomads revolted, but because the students and middle classes of Addis Ababa took up the cause (Clapham, 1990). Selassie became infamous for his efforts to deny the 1972–73 famine, but it was the last in a succession of failures to address famines, which in Tigray and Wollo in the 1950s and 1960s had been:

treated with official indifference, bordering on hostility towards the peasants who were considered sufficiently ungrateful for the divinely‐sanctioned rule of Haile Selassie as to allow themselves to defame his reputation by dying of famine (de Waal, 1991, p. 58, drawing on work by Mesfin Wolde Mariam).

The separatist Tigrayan People's Liberation Front also grew out of the peasant revolts following the 1973 famine (de Waal, 1991); the Tigray famine of the 2020s may thus be seen in part as a continuation of an older story (Weldemichel, 2022). The indifference of Selassie to the plight of his subjects contrasts with Mujib's efforts to attract aid for Bangladesh in 1974, when he begged the international community for assistance in a speech at the UN.

Famines may also directly affect the balance of political power if contending elites recognise that the crisis creates the possibility for regime change. Notably, the Great Famine in Ireland helped a fractious political class towards a more unified articulation of common interests against Britain (Kinealy, 1994). In China, grassroots cadres were dissatisfied with the moderate reforms after the Great Leap Forward famine, while Communist Party leaders like Deng Xiaoping were disgusted by Mao's failures to acknowledge the famine, weakening his hold on the party leadership (Becker, 1996). The 1973 famine in Ethiopia was implicated in the loss of peasant, middle class, and student support for Selassie's empire (Clapham, 1990; see also Shepherd, 1975; de Waal, 1991).

Revolution, as in Ethiopia, is rare after a major famine. But before the floods started in June 1974, Bangladesh was already in ferment, and hunger, inflation, and corruption were high on the political agenda. The left (the Jatiya Samajtantrik Dal and the United Front) rallied against the ruling party, besieged offices tasked with distributing relief, and campaigned for universal foodgrain rations and the tackling of corruption, smuggling, and speculation (Maniruzzaman, 1975b, p. 121). The economic and food crises were blamed on smuggling to India, and the regime was criticised as pro‐India (Khondker, 1985). The government took an aggressive stance against the far left (Maniruzzaman, 1975a); thousands of Awami League workers were killed by the regime's opponents, and leftists were jailed, killed, or driven underground (Karim, 2005).

Disillusion spread across middle‐ and upper‐class idealists who had joined forces with the peasant classes to fight for an egalitarian ‘Golden Bengal’ only a couple of years previously. Former Planning Commission member (and famine researcher) Anisur Rahman (quoted in Tripathi, 2014, p. 234) reflected that:

The war and its aftermath were painful not only because of what happened, but because of the dream that has been shattered… And we lost that dream to a great extent because of the betrayal of the so‐called nationalist elites… We ate together, starved together, suffered together and shared our lives. [But after independence,] [t]he elite rejected the people.

Criticism of Mujib personally had been muted until the famine, but ‘the people now cursed not only the government but also Sheikh Mujib himself’ (Mascarenhas, 1986, p. 44); ‘[t]he year of the famine became the pivot of Mujib's decline’ (Lifschultz and Bird, 1979, p. 46). The emotional quality reported of public disillusionment suggests his fall was painful precisely because he had been so beloved (and as Father, symbolically and literally responsible for feeding them).

The Awami League's loss of legitimacy did not lead to a mass political uprising, but the series of coups after the famine were influenced by the expectation that such an uprising was possible. Fearful for his control of power, Mujib changed the constitution to establish a single‐party regime in early 1975, with himself as president. A coup by junior army officers resulted in the assassination of Mujib and his family in August 1975, and there was a notable lack of public grieving after this brutal mass murder (Tripathi, 2014, p. 250):

By afternoon, the villagers' initial anxiety had given way to euphoria. ‘No one is crying for Mujib,’ [one woman] told us. ‘He has got his due.’ There was almost an air of celebration in [the village], as groups of villagers gathered to discuss the news (Hartmann and Boyce, 1983, p. 240).

Contemporary accounts indicate that Mujib had been personally distressed by the famine and made few public appearances that year. He told a UN official: ‘Country is fighting for survival. I am fighting for survival’ (Gerlach, 2010, p. 168). Unlike Selassie or Mao, Mujib made little attempt to conceal or downplay the disaster, begging the international community for assistance. This does not absolve Mujib for the failures of his administration, but it does indicate a sense of moral responsibility which distinguishes the 1974 famine from others: the regime leadership was aware that the famine was politically costly, and had historical and social affinities with the starving masses.^4^ No such moral responsibility or affinity is detected among those who ruled over the Irish, Bengal (1943–44), or Ethiopian famines, least of all in imperial British attitudes to the ‘late Victorian holocausts’ (Davis, 2001).

### 4.2 Policy and institutional change

A series of post‐famine ruptures in public policy shifted Bangladesh on to a development pathway from which no subsequent government has substantially deviated. A consensus about the essentials of development policy emerged among political, bureaucratic, and business elites (Hossain, 2017). Parallel cognitive breaks can be seen in the ruling elites of other post‐famine polities. The Great Leap Forward famine transformed the Chinese development project by wreaking ‘drastic cognitive changes … among both elites and masses’ with respect to the radical collectivisation that had caused the catastrophe (Yang, 1996, p. 240). In Bangladesh, the brief flirtation with socialism ended; ‘the ideology of the state emerged as a distinct form of “Bangladeshi” nationalism in which beating the “basket case” label was a motive force’ (Hossain, 2017, p. 131).

The first rupture with the left‐leaning policies of liberation was the government's capitulation to donor pressures for devaluation, monetary stabilisation, cuts in public subsidies, and market‐friendlier policies (Sobhan, 1982). In April 1975, the government devalued the currency, having already agreed to reform investment policy. Donors kept ‘Bangladesh twisting in the wind’ through 1974 (Sobhan, 1982, p. 193), cutting down aid commitments during its crisis.

The second rupture came with the government of General Ziaur Rahman, on whose rule aid donors looked more favourably (Sobhan, 1982, p. 196). Zia established an institutional culture of military–bureaucratic rule, repressed opposition and dissent, changed the constitution to reject socialism and secularism, opened the country's arms to Western and Middle Eastern donors, and reoriented economic and social policy towards market competition with some pro‐poor interventions. The regime brought some economic and political stability in a period with fewer ecological and economic shocks. When Zia, too, was assassinated in 1981, he was replaced by General Hussain Muhammad Ershad who ruled until he was toppled by a democratic uprising in 1990. By then, the foundations of the national development project were established.

Many within the political and policy elite recognised that economic reforms were necessary (Sobhan, 1982), yet they resisted World Bank prescriptions to privatise public grain procurement and cut subsidies out of fear of another famine (Chowdhury, 1986, 1988; Adams, Jr., 1998). Averting famine ‘remained uppermost in the minds of donor programmers and government policymakers for the next 20 years’, with management of public food stocks central to the policy agenda (Atwood et al., 2000, p. 153). Procurement and pricing policies helped to increase production and stabilise prices, while redirecting food to the most vulnerable. The system was geared towards responding to shocks through price monitoring, food assistance, and deregulating foodgrain trade (Ahmed, Haggblade, and Chowdhury, 2000; Islam, 2012; Raihan, 2013; Murshid, 2022).

### 4.3 Social relations

Relations between those who rule and those who starve during a famine are irrevocably altered. Ireland lost half of its population to death or migration, but the remainder became guinea pigs for the testing of Malthusian ideas about health, poor relief, and the capitalist restructuring of agrarian relations (Nally, 2008). Communist famines ‘helped the regimes to enforce the collective order’, paving the way to force through industrial and other policies (Wemheuer, 2014, p. 248). In China, draconian birth control and internal migration controls were introduced after the Great Leap Forward famine (Wemheuer, 2014), while agrarian and economic reforms were developed with greater caution (Yang, 2012). In the Soviet Union, state policy emphasised living standards after the famine of the early 1930s, and foodgrain imports became more important (Wemheuer, 2014).

In Bangladesh, public policy was reoriented towards rural, agricultural, and food security concerns, reversing the earlier urban bias (Ahmed, Haggblade, and Chowdhury, 2000). This also included a reorientation towards women, in particular poor rural women. The famine demolished any lingering notions that the benevolent patronage of rural elites or the agricultural economy could protect the landless poor (Alamgir, 1980; see also BRAC, 1992, p. 6). Into the breach left by this broken moral economy a new set of actors emerged with financing from charity and international aid sectors. The best‐known are rural credit or microfinance organisations, and leaders of the two most important, BRAC and the Grameen Bank, both cut their teeth on famine relief. Professor Muhammad Yunus of the Grameen Bank recalled:

The year 1974 was the year which shook me to the core of my being. Bangladesh fell into the grips of a famine… They were everywhere. You couldn't be sure who was alive and who was dead… The government opened gruel kitchens to bring people to specified places in town. But every new gruel kitchen turned out to have much less capacity than was needed… I started to feel useless in the face of so many starving people pouring into Dhaka. Social organizations set up feeding centres in various parts of the city (Yunus and Jolis, 1999, pp. 3–5).

Yunus began to question the value of economic theory, and to consider practical responses. Fazle Hasan Abed, the founder of BRAC, similarly cited the 1974 famine as a turning point during which he learned that poverty reduction efforts needed to work directly with women. These are lessons that have remained central to BRAC's strategy to date, influencing successive generations of Bangladeshi policymakers (Hossain, 2017).

### 4.4 The incentives and capacity to prevent famine after 1974

The 1974 famine in Bangladesh renewed political commitment to preventing such events and mitigating extreme hunger, incentivised by the decimation of the regime that failed to prevent the crisis. It was not a mass uprising, but a coup by junior officers, aware of the loss of legitimacy of the regime and its new vulnerability, that took the life of Mujib, the once popular leader, and removed his party from power. Political commitment to tackle famine subsequently grew at the national and international levels, and entailed the improvement of relations between them, visible in the growth of aid following 1974. The drastic effects of the famine also incentivised political elites at the national and international levels to invest in state capacity to improve food security and disaster response. State institutions to prevent and respond to famine were insulated against routine domestic and international politics, and they have endured.

## 5 CONCLUDING DISCUSSION

The 1974 famine was the first and last such episode in independent Bangladesh, even though it remained poor, food‐insecure, and acutely vulnerable to ecological and global economic crises in subsequent decades. In attempting to show why the country prevented famines after 1974, this article has drawn on the literature on the politics of famine, which draws attention specifically to the political incentives (regardless of regime types) and institutional capacities (regardless of levels of development) to mitigate (in the short term) and prevent (in the medium term) such catastrophes.

This account of the Bangladesh episode in 1974 enables us to revisit and refine political theories of famine, pointing to the need to explore not only the political dimensions of the causes of famine, which receive significant attention, but also the political effects of famine, which do not. As the article shows, these can result in enduring institutional arrangements and political incentives to prevent a reoccurrence, including agreements of the kind that de Waal calls ‘anti‐famine social contracts’. The Bangladesh case confirms the findings of Banik and Rubin that democratic institutions and political competition are insufficient to prevent famine; a popularly‐elected party facing threats from the left was not motivated to reallocate foodgrains to those who needed them most. As Banik has highlighted in relation to Orissa, political competition may in fact have made it more likely that the focus of emergency relief would remain the urban and middle class. In its aftermath, the Bangladesh case supports arguments made by de Waal about an anti‐famine social contract: the catastrophe wrought cognitive change among key members of the elite and a shared commitment to investment in famine prevention. An important feature of the Bangladesh story is the delayed and reluctant release of food aid by the US, which was very likely influenced by ideological factors regarding the economic unviability of the devastated new nation.

Three features of the Bangladesh example stand out as relevant to theorising the politics of famine. First, as the selectorate theorists point out, aid‐dependent countries rely on assistance to help them relieve famines, but even then, the choice of who gets the food aid will depend on which groups are relevant to the balance of power (Plümper and Neumayer, 2009). In 1974, these politically relevant groups were not the starving rural landless.

Second, as the political ecology, biopolitics, and empire approaches to famine and the ‘new famine politics’ theories of twenty‐first century famines have argued, key political actors are supra‐national: the Bangladesh case supports the view that more needs to be understood regarding the political incentives for international actors to intervene—or to withhold action—during a famine. The famines in Palestine, Ethiopia, and Sudan in the mid‐2020s point to weak international and regional political incentives to intervene to prevent campaigns of ethnic cleansing, as well as to active complicity in or support for crimes of mass starvation. Given the swingeing cuts in US development aid in 2025, the international community also has fewer resources for food aid or other humanitarian assistance, giving it greater scope for exercising discretion with respect to who receives life‐saving assistance, and on what terms. A later paper will address the role of the international community, in particular the US, in delaying, in effect, withholding, food aid for Bangladesh in 1974.

Third, the Bangladesh case draws attention to a singular political effect at the heart of the move towards an anti‐famine contract: the loss of legitimacy by the ruling group, marked by the brutal murder of a political leader who was until recently a beloved national hero. The power of this dramatic loss of legitimacy in terms of committing the ruling class to a reversal of policy direction and prioritising food security is notable. The loss of political legitimacy may not lead to mass uprisings against rulers during famine, but it weakens their power base and leaves them vulnerable to a challenge.

## CONFLICT OF INTEREST STATEMENT

No conflict of interest reported.

## Data Availability

Data sharing not applicable to this article as no datasets were generated or analysed during the current study.

## Endnotes

^1^
The literature explaining Bangladesh's food security achievements is broadly in agreement about the policies and programmes that contributed. The source of record is the 2000 volume titled *Out of the Shadow of Famine*, comprising authors from Bangladeshi academic and policy institutions and the International Food Policy Research Institute and other researchers (Ahmed, Haggblade, and Chowdhury, 2000; see also Murshid, 2022).

^2^
The 1974 census figures come from the Bangladesh Bureau of Statistics.

^3^
See https://en.wikisource.org/wiki/Address_to_the_United_Nations_General_Assembly_(Sheikh_Mujib,_1974‐09‐25) (last accessed on 12 January 2026).

^4^
These come through in the pained writing by elites about this time. A particularly thoughtful account of 1974 is by the former head of the Planning Commission, Nurul Islam (2003).
